# Identification and characterization of nanobodies specifically against African swine fever virus major capsid protein p72

**DOI:** 10.3389/fmicb.2022.1017792

**Published:** 2022-10-13

**Authors:** Jifei Yang, Mengyao Jing, Qingli Niu, Jinming Wang, Yaru Zhao, Meng Liu, Guiquan Guan, Jianxun Luo, Hong Yin, Zhijie Liu

**Affiliations:** ^1^African Swine Fever Regional Laboratory of China (Lanzhou), State Key Laboratory of Veterinary Etiological Biology, Lanzhou Veterinary Research Institute, Chinese Academy of Agricultural Sciences, Lanzhou, Gansu, China; ^2^China Agricultural VET. BIO. Science and Technology Co, Ltd, Lanzhou, China; ^3^Animal Husbandry and Veterinary Bureau of Dingxi City, Dingxi, Gansu, China; ^4^Jiangsu Co-Innovation Center for the Prevention and Control of Important Animal Infectious Disease and Zoonosis, Yangzhou University, Yangzhou, China; ^5^Institute of Special Animal and Plant Sciences, Chinese Academy of Agricultural Sciences, Changchun, China

**Keywords:** African swine fever, major capsid protein p72, phage display, nanobody, biosensor

## Abstract

African swine fever virus (ASFV) is a large and very complex DNA virus. The major capsid protein p72 is the most predominant structural protein and constitutes the outmost icosahedral capsid of the virion. In the present study, the nanobodies against ASFV p72 protein were screened from a camelid immune VHH library by phage display technique. Nine distinct nanobodies were identified according to the amino acid sequences of the complementary determining regions (CDRs), and contain typical amino acid substitutions in the framework region 2 (FR2). Six nanobodies were successfully expressed in *E*. *coli*, and their specificity and affinity to p72 protein were further evaluated. The results showed that nanobodies Nb25 had the best affinity to both recombinant and native p72 protein of ASFV. The Nb25 possesses an extremely long CDR3 with 23 amino acids compared with other nanobodies, which may allow this nanobody to access the hidden epitopes of target antigen. Furthermore, the Nb25 can specifically recognize the virus particles captured by polyclonal antibody against ASFV in a sandwich immunoassay, and its application as a biosensor to target virus in PAM cells was verified by an immunofluorescence assay. Nanobodies have been proven to possess many favorable properties with small size, high affinity and specificity, easier to produce, low costs and deep tissue penetration that make them suitable for various biotechnological applications. These findings suggest that nanobody Nb25 identified herein could be a valuable alternative tool and has potential applications in diagnostic and basic research on ASFV.

## Introduction

African swine fever (ASF) is one of the most devastating infectious diseases of domestic pigs and wild boar caused by African swine fever virus (ASFV) (Dixon et al., 2020). The disease is characterized by highly contagious, acute, hemorrhagic fever and exceptionally high lethality (Gallardo et al., 2019). Although the causative agent is very host specific and without zoonotic potential, the frequent outbreaks and the continue spread of the disease have caused serious socio-economic consequences globally (Ata et al., 2022). Since its identification in Georgia in 2007, the dispersal of ASF was accelerated and the situation worsened deeply after the outbreaks occurred in China in 2018 (Zhou et al., 2018; Dixon et al., 2020). Nowadays, continual outbreaks and spread of ASF are taking place in Europe and Asia. More recently, the disease was reintroduced into the Dominican Republic in July 2021 and later Haiti after its initial emergence in the Western hemisphere over 40 years (Gonzales et al., 2021; Urbano and Ferreira, 2022). In the last decade, great efforts and notable advances have been made on the vaccine development of ASF, especially for the live attenuated vaccines (LAVs) (Urbano and Ferreira, 2022). Previous attempts on the inactivated vaccines against ASFV have failed to induce protection (Dixon et al., 2020). Some viral antigens of ASFV have been evaluated and applied in the development of subunit, DNA and virus-vectored vaccines, which provide a variable degree of protection against challenge with ASFV (Urbano and Ferreira, 2022). Currently, several LAVs containing one or more deletion of virulence-associated genes in the genome have been reported and showed to confer fully protection during challenge, such as ASFV-G-Δ9GL/ΔUK, HLj/18-7GD and ASFV-G-ΔI177L (O’Donnell et al., 2017; Borca et al., 2020; Chen et al., 2020). Although LAVs seem to be the promising vaccine candidates against ASFV so far (Urbano and Ferreira, 2022); their safety, genetic stability and side effects in animals should be fully considered and evaluated prior to large-scale field application.

ASFV is the only known DNA arbovirus, the soft ticks of the genus *Ornithodoros* could serve as reservoirs and biological vectors, and the vector competences of *Ornithodoros moubata* in the sylvatic transmission cycle in Africa and *Ornithodoros erraticus* in Europe have been well documented (Plowright et al., 1974; Boinas et al., 2011). As the solely member of the *Asfivirus* genus of *Asfarviridae* family, ASFV is a large enveloped virus with 170 to 190 kb double stranded DNA genome, which contains more than 150 open reading frames (ORFs) and encodes 150 ~ 200 viral proteins, depending on virus isolates (de Villiers et al., 2010; Dixon et al., 2013; Alejo et al., 2018). Currently, 24 genotypes of ASFV have been identified based on the *B646L* gene, and the highly virulent genotype II is the major one circulating outside of Africa since its introduction into Caucasus in 2007 (Quembo et al., 2018; Ata et al., 2022).

Despite great efforts and decades of research, the functions of many ASFV-encoded proteins have not been disclosed yet (Gaudreault et al., 2020). The basic research relevant to virus invasion, replication and pathogenesis mechanisms is vital for the development of effective vaccine and other control measures of ASF. To date, 68 structural proteins of ASFV have been identified by mass spectrometry (Alejo et al., 2018). Among them, p72 is the most predominant structural protein and comprises 31 ~ 33% of the total protein of the virion (Carrascosa et al., 1984). It constitutes the outmost icosahedral capsid and is a high immunogenic viral protein, which has been efficiently used as an antigen for diagnostic purpose (Yu et al., 1996). Moreover, the p72 major capsid protein has also been proven to be involved in the virus attachment to target cells (Gomez Puertas et al., 1996). Recently, the high-resolution cryo-EM structure of the p72 protein has been reported and the results revealed that three p72 molecules form a thermostable trimer with the assistance of B602L (Liu et al., 2019).

Nanobodies, also referred to single-domain antibodies, are derived from heavy-chain antibodies (HCAbs) that naturally occurring in sera of camelids and lacking light chains. As a novel type of antibody fragment, nanobody has attracted extensive interest and is expected to be applied as a potential tool in the research and biomedical fields in the past two decades (Hamers-Casterman et al., 1993). Compared with conventional antibodies, nanobodies have been shown to possess many striking properties, including high affinity and specificity, small molecular size, economical and easy production, deep tissue penetration and recognition of hidden epitopes (Muyldermans, 2013; Salvador et al., 2019). Due to the beneficial properties, an increasing number of nanobodies have been explored and used in a wide range of routine and innovative applications for diagnostics and therapeutics (Salvador et al., 2019).

In this study, specific nanobodies against ASFV p72 protein were screened from a camelid VHH library by phage display technique. The identified nanobodies could be used as valuable alternative tools in diagnostic and research purposes on ASFV.

## Materials and methods

### Biosafety and ethics statements

All experiments involving ASFV were conducted under biosafety level 3 (BSL-3) facilities in Lanzhou Veterinary Research Institute (LVRI) of Chinese Academy of Agricultural Sciences (CAAS), and were accredited by China National Accreditation Service for Conformity Assessment (CNAS) and the Ministry of Agriculture and Rural Affairs of China. The animal treatments and sample collection were performed in accordance with the Animal Ethics Procedures and Guidelines and have been approved by the Animal Ethics Committee of LVRI, CAAS.

### Cells, virus, and samples

The primary porcine alveolar macrophages (PAMs) were isolated by lung lavage from 2 months old healthy pig tested negative for ASFV, classical swine fever virus (CSFV), porcine reproductive and respiratory syndrome virus (PRRSV), pseudorabies virus (PRV), porcine parvovirus (PPV) and porcine circovirus type 2 (PCV2). PAM cells were cultured in RPMI 1640 medium (Gibco, United States) containing 10% (*v*/*v*) fetal bovine serum (FBS; Gibco, United States) and antibiotics (100 units/ml penicillin and 100 mg/ml streptomycin) at 37°C in a 5% CO_2_ incubator, as described previously (Malmquist and Hay, 1960). ASFV genotype II strain CN/SC/2019 and the positive sera collected from surviving pigs naturally infected by virus were provided by African Swine Fever Regional Laboratory of China (Lanzhou).

### Phage display VHH library and bio-panning

The phage display library containing VHHs raised against ASFV p72 protein (GenBank: FR682468) was previously constructed from peripheral blood lymphocytes of immunized Bactrian camels (*Camelus bactrianus*) in our laboratory, with a library capacity of 2.2 × 10^8^ cfu/ml. The generated VHH library exhibited high capacity with a correct insert rate of 91.2% and rich sequence diversity of complementary determining regions (CDRs) determined by colony PCR and sequencing (Zhao, 2019). The nanobodies specifically against ASFV p72 protein were screened from the generated phage display library by bio-panning as described previously (Sheng et al., 2019). After rescued by helper phage M13K07, three consecutive rounds of bio-panning were carried out on solid phase coated p72 antigen. Briefly, the recombinant p72 protein was coated onto a microtiter plate at a concentration of 10 μg/ml and blocked with 5% skim milk in PBST (PBST containing 0.05% Tween 20). Antigen free wells were set up for the blank control. In order to eliminate no specific binding, the recombinant phage particles were primary incubated with 2% skim milk for 30 min, and then added to the blocked wells and incubated with coated antigen for 1 h. After washing nine time with 0.1% PBST (PBS containing 0.1% Tween), the bound phages were eluted from the plate and used to infect exponential *E. coli* TG1 cells for amplification and titration. The amplified phages were used in the following round of bio-panning. Decreased antigen on coated plate (5 μg/ml and 2 μg/ml) and intensive washing buffer with increased concentrations of Tween 20 in PBST (0.2 and 0.3% PBST) were used in the second and third round of bio-panning to remove the off-target and weak binders, respectively. The enrichments for antigen-specific phages of each round of bio-panning were assessed according to the number of output and input phages.

### Phage ELISA

After three rounds of bio-panning, 40 individual colonies were randomly selected and subjected to monoclonal phage ELISA to identify the binders with p72 protein as previous described (Yang et al., 2014). Briefly, clones were cultured in 2 × YT-AG medium (supplemented with 100 μg/ml ampicillin and 1% glucose) to OD_600_ reached 0.4 ~ 0.5 and infected with M13K07 helper phage. A microliter plate was coated with 2.5 μg/ml recombinant p72 protein overnight at 4°C, and PBS was used as a negative control. After washing three times with PBST, the plate was blocked with 2.5% (w/v) skim milk in PBS for 2 h at room temperature (RT). Then, recombinant phages were incubated in the antigen coated microliter plate for 2 h at RT. The plate was washed six times, and the rabbit anti-M13 IgG was added to each well and incubated for 1 h at 37°C. The plate was washed again, and the goat anti-rabbit IgG conjugated to HRP was added and incubated for 1 h at 37°C. After another washing step, the colorimetric reaction was developed by adding of o-phenylenediamine dihydrochloride (OPD) substrate solution and incubated for 30 min at RT. The reaction was stopped by 3 M H_2_SO_4_ and the absorbance at 490 nm was measured by a microtiter plate reader (Thermofisher, United States). Colonies were considered to be positive when their absorbance was 3-fold more than that of negative control. The plasmids from positive colonies were sequenced (Sangon Biotech Co. Ltd., Shanghai, China) to identify different nanobodies.

### Expression and purification of nanobodies

The identified VHH genes of positive clones were amplified by PCR with primers VHH-F (5′-CGGAATTCGATGTGCAGCTGGTGGAGTCT-3′) and VHH-R (5′- TGCTCGAGTGAGGAGACAGTGACCTGGGTCC-3′). They were subcloned into the expression vector pET-28a by *EcoR* I and *Xho* I restriction enzyme sites. After confirmed by PCR and sequencing, the recombinant plasmids were transformed into the *E*. *coli* BL21 (DE3) competent cells. Then, the *E*. *coli* cells were cultured in terrific broth medium (containing 50 μg/ml kanamycin) and incubated at 37°C with shaking at 200 rpm/min. Once the optical density reached 0.6 ~ 0.8, the cultures were induced using *isopropy β-D-thiogalactopyranoside* (IPTG) at a final concentration of 0.4 mM and incubated for 7 ~ 9 h at 37°C with shaking at 220 rpm. The cells were harvested by 8,000 × g centrifugation for 10 min. Cell pellet was resuspended in PBS and disrupted by ultrasonication on ice bath. The expressed nanobodies were purified using affinity chromatography on nickel column (GE Healthcare, United States). The purified nanobodies were further identified by sodium dodecyl sulfate polyacrylamide gel electrophoresis (SDS-PAGE) and quantified by Qubit 2.0 fluorometer using Qubit Protein Assay Kit (Qubit, Thermo Fisher Scientific) according to the protocols.

### The reactivity of isolated nanobodies

The reactivity of nanobodies was analyzed by an indirect ELISA. The microtiter plate was coated with 100 μl (1 μg/ml) nanobodies diluted in carbonate buffer and incubated at 4°C overnight. After washing three times with PBST, 100 μl (5 μg/ml) of ASFV p72 protein was added to each well and incubated for 1 h at 37°C. The plate was washed and blocked with 1% BSA in PBST for 1 h at 37°C. Then, the ASFV positive and negative swine sera (diluted 1:100 in PBST) were added and incubated for 1 h at 37°C. The plate was washed and 100 μl of HRP-conjugated rabbit anti-pig antibody (diluted 1:20,000 in PBST, sigma, United States) was added and incubated for 1 h at 37°C. After another washing step, 100 μl of TMB substrate was added to develop the color reactions. Afterward, the reaction was terminated with 0.3 M H_2_SO_4_ and the absorbance at 450 nm was measured by a microtiter plate reader (Thermofisher, United States). All assays were carried out in duplicate.

### The specificity of isolated nanobodies

Primary PAMs were inoculated with ASFV at a multiplicity of infection (MOI) of 1.0. The cells and supernatants were collected at 96 h post-infection and then lysed by freeze–thaw cycles. Cell debris was removed by centrifugation at 4°C and 12,000 × g for 15 min and cell-free supernatant containing viral particles was collected for further analysis. The IgG purified from ASFV-positive swine sera using Protein A-immunomagnetic beads (Sangon Biotech Co. Ltd., Shanghai, China) was coated (1:1000 diluted in carbonate buffer) on the microtiter plate and incubated at 4°C overnight. The plate was washed three time with PBST, and the diluted virus stock (1:20 in PBS) was added and incubated for 1 h at 37°C. The plate was washed and blocked with 1% BSA in PBST for 1 h at 37°C. Then, nanobodies was added and incubated for 1 h at 37°C, and PBS was used as a negative control. The plate was washed again and incubated with HRP-conjugated rabbit anti-His IgG (diluted 1:15,000 in PBST, Abcam, United Kingdom) for 1 h at 37°C. After another washing step, the colorimetric reaction was developed and measured as aforementioned. All assays were carried out in duplicate.

### Western blot analysis

Primary PAMs infected with ASFV at an MOI of 1.0 were collected at 96 h post-infection. The cell lysates were separated by SDS-PAGE and transferred to a polyvinylidene fluoride (PVDF) membrane. The membrane was blocked with 5% skim milk in TBST (Tris-buffered saline containing 0.05% Tween 20) at 4°C overnight. The membrane was washed three times and incubated with nanobodies for 1 h at RT. Then, the membrane was washed and incubated with HRP-conjugated rabbit anti-His IgG (diluted 1:2,000, Abcam, United Kingdom) for 1 h at RT. After washed again, the immunoreactive bands were visualized with a chemiluminescent substrate (ECL; Thermo-Fisher, United States).

### Immunofluorescence assay

Nanobodies were conjugated with fluorescein isothiocyanate (FITC) using a FITC Conjugation Kit (Bioss, China) according to the manufacturer’s instructions. Primary PAMs were seeded in glass bottom cell culture dishes (NEST, China) at a density of 4.0 × 10^6^ cells and infected with ASFV at an MOI of 1.0. At 48 h post-infection, ASFV-infected or negative control cells were fixed with 4% paraformaldehyde for 10 min, permeabilized in 0.1% Triton-X 100 for 10 min, and blocked with 5% BSA for 1 h at RT. The fixed ASFV-free and -infected PAMs were washed three times with PBS and incubated with FITC-conjugated nanobodies for 1 h at 37°C. Moreover, ASFV-infected PAMs were also incubated with ASFV-positive and -negative serum for 1 h at 37°C, respectively. After washing three times with PBS, the FITC-conjugated rabbit anti-pig IgG (Sigma, United States) was added and incubated for 1 h at 37°C. Then, cells were incubated with DAPI (Invitrogen, United States) for 2 min at RT and rinsed with PBS. The cells were visualized for immunofluorescence with a fluorescence microscope (Leica, Germany).

## Results

### Bio-panning and phage ELISA analysis

**Figure 1:**
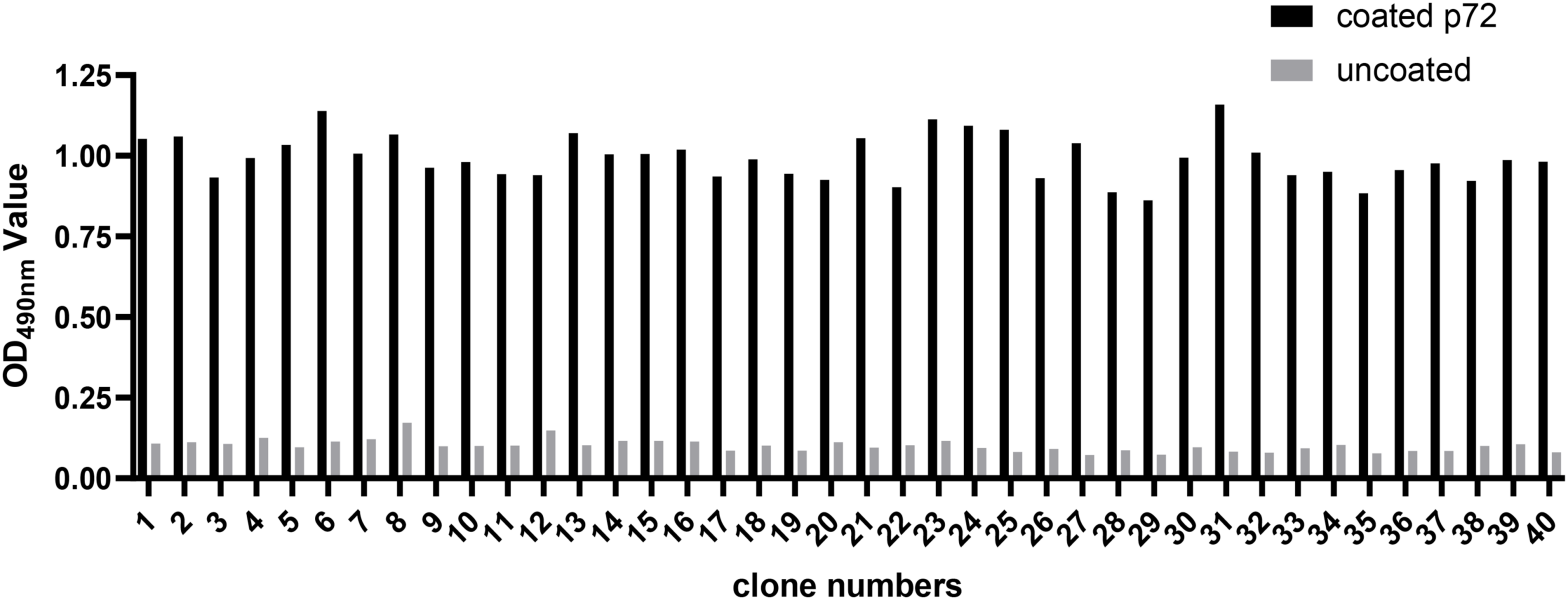
Identification of ASFV p72 protein binding phages by monoclonal phage ELISA. Forty clones selected from final round bio-panning were detected, and all of them were identified as positive. The clone was regarded as positive if the absorbance in the antigen-coated well was at least threefold greater than that in uncoated well.

In the present study, three consecutive rounds of bio-panning were performed to isolate the nanobodies specific for ASFV p72 protein from a phage display VHH library. The enrichment of phages of each round of bio-panning was evaluated and the results showed that the recombinant phage particles were significantly enriched during the bio-panning (Table 1). In order to verify whether the enriched phages contained specific VHHs against ASFV-p72 protein, 40 clones were selected from the final round of bio-panning and subjected to single-phage ELISA for further identification of specific binders. The results revealed that all selected clones were positive and showed a good target recognition with binding ratios in contrast to blank control of greater than 3.0 (Figure 1).

**Table 1:** The enrichment of the nanobodies in the phage display library as detected through three rounds of bio-panning.

Round	Input	Output	Enriching factor (input / output)
1st	3.80 × 10^11^	1.66 × 10^5^	2.29 × 10^6^
2nd	1.93 × 10^12^	1.40 × 10^8^	1.38 × 10^4^
3rd	2.49 × 10^11^	3.59 × 10^8^	6.94 × 10^2^

### Alignment of VHH amino acid sequences

**Figure 2:**
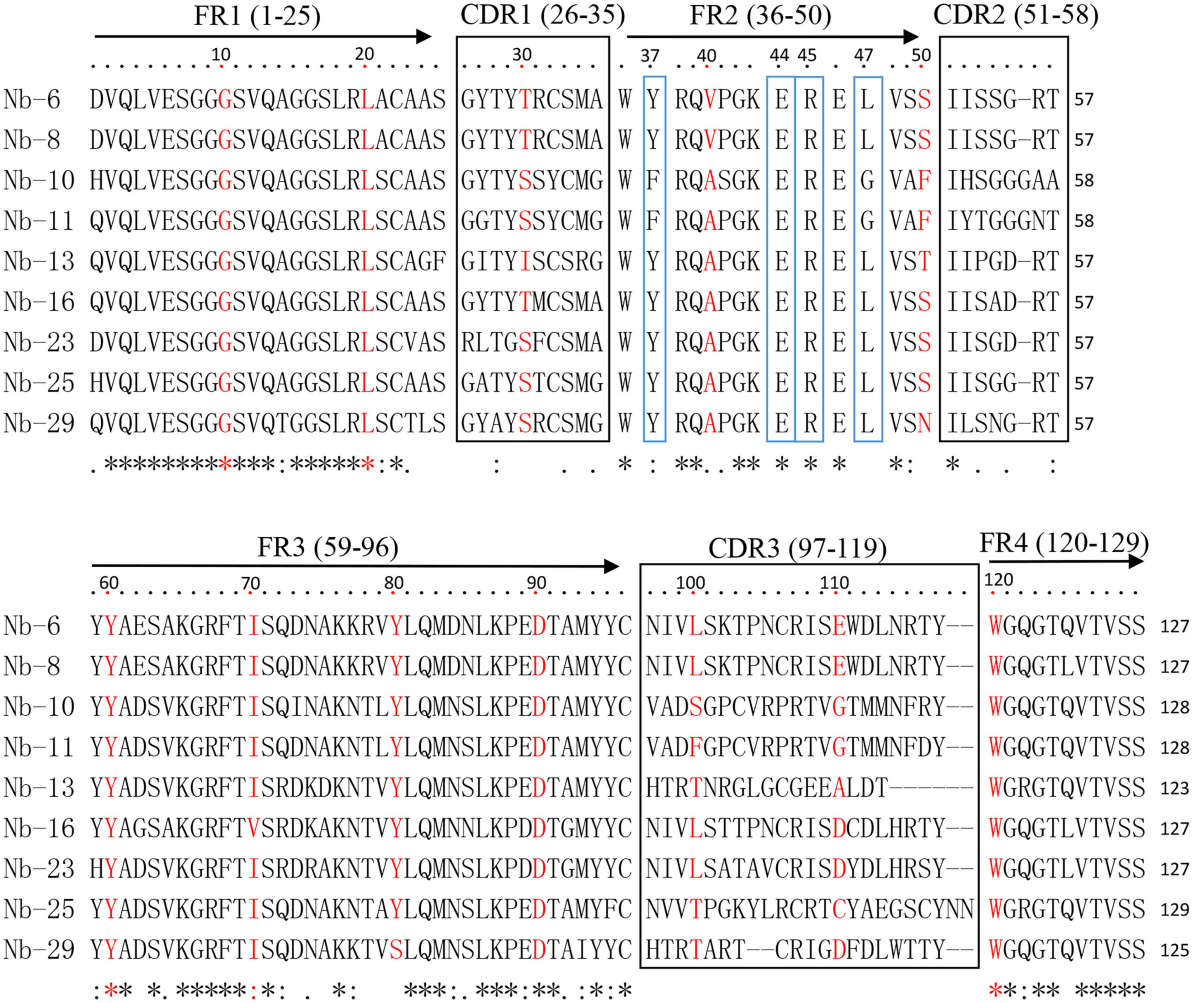
Alignment of amino acid sequence of nanobodies against ASFV p72 protein. The sequences are grouped according to their CDRs. The residues at positions 37, 44, 45, and 47 in FR2 are indicated by blue boxes.

All 40 clones were subsequently sequenced and showed to contain the correct framework regions of VHH fragments. Sequence analysis revealed that nine distinct nanobodies were obtained based on the amino acid sequence classification of the complementary determining regions (CDRs). These nanobodies were designated as Nb6, Nb8, Nb10, Nb11, Nb13, Nb16, Nb23, Nb25 and Nb29, respectively (Figure 2). Among them, typical amino acid substitutions were observed in the framework region 2 (FR2), including G/E and L/R substitutions at positions 44 and 45 of all nanobodies, V/Y and W/L substitutions at positions 37 and 47 of seven nanobodies (Nb6, Nb8, Nb13, Nb16, Nb23, Nb25, and Nb29), and V/F and W/G substitutions at positions 37 and 47 of two nanobodies (Nb10 and Nb11), respectively (Figure 2). These results indicated that they are camelid-derived heavy-chain-only antibodies. Moreover, these nanobodies possess especially long CDR3, which contain 17–23 amino acids and exhibit high sequence variability (Figure 2).

### Expression and purification of nanobodies

**Figure 3:**
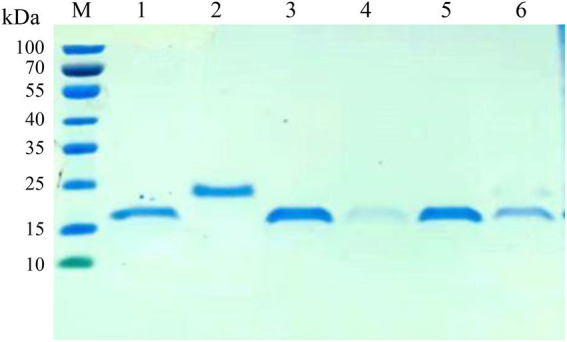
SDS-PAGE analysis of the purification of nanobodies. M: protein molecular marker; lane 1: The purified Nb6; lane 2: Nb8; lane 3: Nb10; lane 4: Nb13; lane 5: Nb23; lane 6: Nb25.

The VHH sequences identified from the positive clones were inserted into the expression vector pET-28a, and transformed into *E*. *coli*. After IPTG induction, six nanobodies (Nb6, Nb8, Nb10, Nb13, Nb23 and Nb25) possess hypervariable CDR3 were successfully expressed with a His-tag. The purification of nanobodies were performed using Ni-NTA affinity column and analyzed by SDS-PAGE. The results showed that the nanobodies were isolated with high purity and exhibited specific band with approximately 20 kDa, which is consistent with the expected molecular size (Figure 3).

### The reactivity of isolated nanobodies

**Figure 4:**
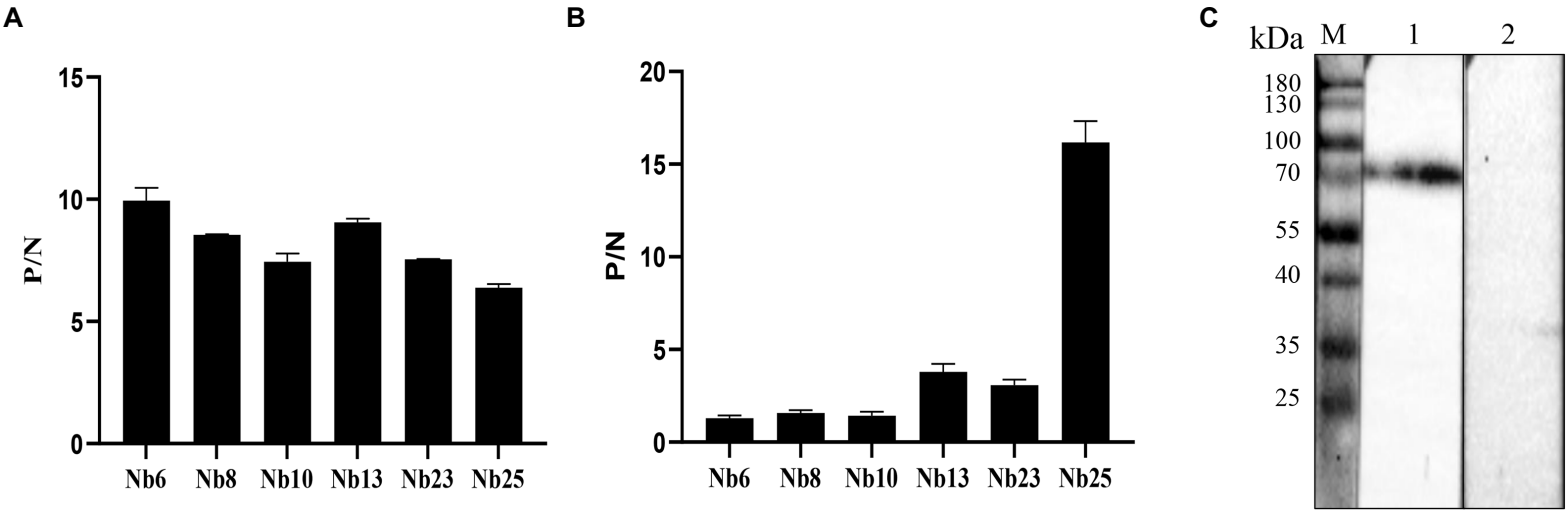
Specificity and affinity of nanobodies with ASFV p72 protein. **(A)** The affinity of nanobodies with recombinant p72 protein analyzed by an indirect ELISA. ASFV-p72 protein was incubated with solid phase coated nanobodies. The ASFV-positive serum and HRP-conjugated rabbit anti-pig antibody were then added. The ASFV-negative serum was used as negative control. The P/N value is the average of two wells. Error bars represent ±SD. **(B)** The affinity of nanobodies with native p72 protein of ASFV analyzed by a sandwich ELISA. The polyclonal antibody against ASFV was coated on the microtiter plate. The diluted virus stock was added to each well. The nanobody was then added, and PBS was used as negative control. The P/N value is the average of two wells. Error bars represent ±SD. **(C)** The specificity of Nb25 analyzed by Western blot. The p72 protein in PVDF membrane was detected by Nb25 and HRP-conjugated rabbit anti-His IgG. M: protein molecular marker; lane 1: PAM cells inoculated with ASFV; lane 2: Negative control.

The affinities of the six nanobodies bind with ASFV-p72 protein were assessed by ELISA. ASFV-p72 protein was incubated with solid phase coated nanobodies as capture reagents, and the immunoreaction was further determined by ASFV-positive swine sera and HRP-conjugated rabbit anti-pig antibody. The results revealed that all six nanobodies could capture ASFV-p72 protein, and high ratios of the absorbance values at OD_450 nm_ between positive and negative sera were observed (Figure 4A). These results suggest that six nanobodies had relatively high affinities to ASFV-p72 protein.

### The specificity of isolated nanobodies

In order to determine whether the nanobodies could bind with native p72 protein of ASFV, a sandwich ELISA was performed. The polyclonal antibody against ASFV was coated on the microtiter plate and applied to capture ASFV particles, and the nanobodies were subsequently allowed to react with viral antigen. In contrast to other nanobodies, Nb25 showed highest binding capacity to native ASFV p72 protein (Figure 4B). The interaction between Nb25 and native ASFV p72 protein was further validated by Western blot. The results showed that Nb25 can specifically recognize the p72 protein of ASFV particles, and excluded the possibility that Nb25 react with His tag of recombinant protein (Figure 4C).

### ASFV imaging in PAM cells with Nb25 probe

**Figure 5:**
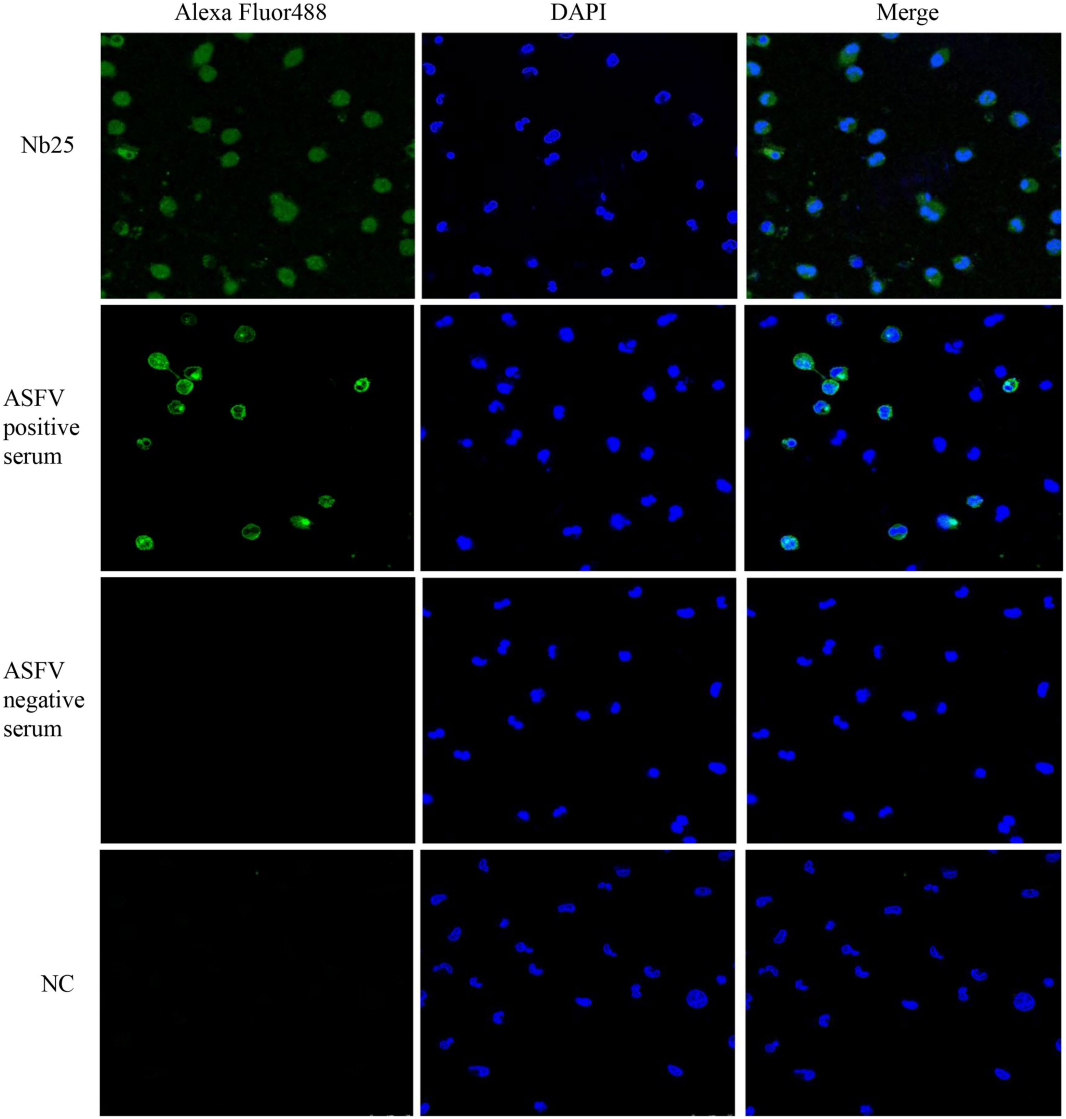
Nb25 as biosensor in imaging ASFV in PAM cells. The PAM cells were immobilized at 48 h post ASFV infection, virus free PAM cells were used as a negative control (NC). ASFV in PAM cells was detected by immunofluorescence assay based on FITC-Nb25, ASFV-positive and -negative serum. ASFV free PAM cells were used as negative control (NC). Nucleus were stained with DAPI.

The PAM cells infected with ASFV were fixed and the presence of virus particles was detected by direct immunofluorescence assay. As shown in Figure 5, ASFV was localized in the cytoplasm after cells were probed with Nb25 conjugated with FITC, and no fluorescence was observed as expected in negative control. The indirect immunofluorescence assay also showed that ASFV can be detected in PAM cells by ASFV-positive serum and the FITC-conjugated rabbit anti-pig IgG (Figure 5). These results suggested that Nb25 can bind with ASFV specifically. As a consequence, Nb25 is a desirable probe to target and visualize ASFV *in vitro* and has potential applications in basic research on ASFV.

## Discussion

Antibodies are very important biological macromolecules that have been widely used for detection, targeting and basic research purposes due to their high affinity and specificity (Salvador et al., 2019). However, the performance of traditional antibodies is still confined in terms of big size, poor penetration and problems in large-scale preparation. As aforementioned, nanobodies exhibit many advantages that could overcome these drawbacks, and make them suitable for various biological applications (Salvador et al., 2019). In the present study, nanobodies against ASFV p72 were screened from the camelid immune VHH library. After three rounds of bio-panning, the recombinant phage particles were enriched obviously, and all selected clones were positive to target protein. These results suggested that the panning process conducted herein is highly efficient and three rounds of panning could sufficiently enrich the recombinant clones containing specific VHHs (Muyldermans, 2013). Ultimately, nine distinct nanobodies specific for p72 protein were retrieved and identified according to their diverse amino acid sequences of CDRs, which are responsible for the antigen binding specificity. Furthermore, these nanobodies possess the hallmark amino acid substitutions at specific sites in the FR2 that involved in the VH/VL interaction of conventional antibodies (Gonzalez-Sapienza et al., 2017).

The molecule of nanobody is 10-fold smaller than conventional antibody (Hamers-Casterman et al., 1993). Owing to its small size, the engineering and recombinant expression of nanobody *in vitro* are readily achieved using prokaryotic and eukaryotic expression systems. In this study, six nanobodies specific to ASFV p72 were expressed in *E*. *coli*, and their affinities bind with target were subsequently verified by ELISA. Although all six nanobodies could bind with recombinant p72 protein with high affinities, their binding affinities to native p72 protein of ASFV varied significantly. This is probably due to the recombinant p72 protein was applied in the bio-panning and screen processes.

Among those nanobodies, Nb25 had the best affinity to native p72 protein determined by ELISA and Western blot. According to the sequence analysis, the Nb25 possess an extremely long CDR3 with 23 amino acids compared with other nanobodies. The long CDR3s are more frequently observed in camelid nanobodies, which can enhance the affinity of nanobodies to target antigens (Nguyen et al., 2000). In addition, the CDR1 and CDR3 of Nb25 contains cysteine residues, the stability of this nanobody might be reinforced by the intermolecular disulfide bridge formed between Cys residues of CDR1 and CDR3. Its small size, extremely long CDR3 and high stability allow Nb25 to better access the concave or hidden epitopes of p72 protein, which may not be accessed by conventional antibodies (Lutje Hulsik et al., 2013; Gonzalez-Sapienza et al., 2017).

Owing to their intrinsic and unique properties, nanobodies have been widely used in the detection of specific causative agents in laboratory tests (Salvador et al., 2019). More recently, several nanobodies against the p54 and K205R protein of ASFV have been isolated from the camelid immune library, they were subsequently applied in the development of nanobody-based cELISA for detection of ASFV antibodies (Zhang et al., 2022; Zhao et al., 2022). In the present study, the nanobodies specific for native p72 protein were selected by a sandwich ELISA, and the Nb25 can specifically recognize the virus particles captured by polyclonal antibody against ASFV. Therefore, the nanobody Nb25 might be served as a potential new diagnostic reagent for detection of ASFV.

The nanobodies exhibit high target specificity and have better tissue penetration than conventional antibodies because of their smaller molecular size, which are suitable for the imaging and investigation of target proteins at the cellular level (Salvador et al., 2019; Bao et al., 2021). As a good tracer, nanobody has been considered to be an attractive alternative for cancer imaging by target tumor specific biomarker (Vaneycken et al., 2011); and it has also been developed as biosensors to target and trace virus in cells, such as foot and mouth disease virus (FMDV) (Wang et al., 2015) and PEDV (Yang et al., 2018). In this study, the immunofluorescence assay revealed that ASFV can be targeted by Nb25 in PAM cells. Therefore, this nanobody could be used as a biosensor for the dynamic tracing of ASFV in living cells, which may be desired to be a promising tool and applied in the basic research related to virus-host interaction in the future.

In conclusion, ASFV p72 specific nanobodies were screened from a camelid immune VHH library by the phage display technique. The nanobody Nb25 was identified and showed high specificity and affinity to both recombinant and native p72 protein of ASFV. Furthermore, the p72-specific Nb25 could be used as a biosensor in tracing, diagnostic applications and basic research on ASFV.

## Data availability statement

The raw data supporting the conclusions of this article will be made available by the authors, without undue reservation.

## Author contributions

JY and ZL: conceptualization, data curation, formal analysis, and funding acquisition. JY and MJ: investigation, methodology, and writing-original draft. JW, QN, and YZ: methodology, formal analysis, clinical sample detection. ML, GG, JL, and HY: formal analysis, review, and editing. All authors contributed to the article and approved the submitted version.

## Funding

This study was financially supported by the National Natural Science Foundation of China (31941012 and 32072830), the Longyuan Youth Innovation and Entrepreneurship Talent Project (2021LQGR24), the Natural Science Foundation of Gansu (21JR7RA018), Gansu Provincial Major Project for science and technology development (20ZD7NA006), ASTIP (CAAS-ASTIP-2016-LVRI), the Jiangsu Co-innovation Center program for Prevention and Control of Important Animal Infectious Diseases and Zoonoses.

## Conflict of interest

YZ was employed by China Agricultural VET. BIO. Science and Technology Co, Ltd.

The remaining authors declare that the research was conducted in the absence of any commercial or financial relationships that could be construed as a potential conflict of interest.

## Publisher’s note

All claims expressed in this article are solely those of the authors and do not necessarily represent those of their affiliated organizations, or those of the publisher, the editors and the reviewers. Any product that may be evaluated in this article, or claim that may be made by its manufacturer, is not guaranteed or endorsed by the publisher.
